# Unexpected results found in larvae samples from two postmortem forensic cases

**DOI:** 10.1007/s11419-021-00601-x

**Published:** 2021-11-06

**Authors:** Olwen Groth, Simon Franz, Helena Fels, Julia Krueger, Gabriele Roider, Torsten Dame, Frank Musshoff, Matthias Graw

**Affiliations:** 1 Institute of Forensic Medicine, University of Munich, Nussbaumstrasse 26, 80336 Munich, Germany; 2 Forensic Toxicological Centre (FTC) Munich, Bayerstrasse 53, 80335 Munich, Germany

**Keywords:** Benzodiazepines, Entomotoxicology, Necrophagous larvae, New psychoactive substances (NPS), Postmortem, Synthetic cannabinoids

## Abstract

**Purpose:**

In forensics, entomological specimens can be used as additional/alternative matrices to detect xenobiotics when human specimens are limited in their application. Despite some advantages over implementing putrefied human remains, most medico-legal laboratories do not include entomotoxicological procedures as routine analytical methods. We thus applied two authentic cases to evaluate necrophagous larvae’s potential as complementary matrices for toxicological analysis after extensive postmortem decomposition.

**Methods:**

Larvae and postmortem human samples, including hair, stomach contents, pericardial fluid, liver, lung, and skeletal muscle, were collected at autopsy. Samples were analyzed by liquid chromatography–tandem mass spectrometry and liquid chromatography–quadrupole time-of-flight mass spectrometry for pharmaceutical substances, illicit drugs, and new psychoactive substances, including synthetic cannabinoids, benzodiazepines, new synthetic opioids, and stimulants.

**Results:**

Nearly all substances detected in human specimens, including several benzodiazepines and synthetic cannabinoids, were also detected in larvae. Surprisingly, some drugs, including the new psychoactive substances EAM-2201 and U-47700, were found exclusively in larvae and hair. The benzodiazepine etizolam was detected only in liver, lungs, and stomach contents, possibly resulting from characteristic tissue distribution in humans and/or larvae.

**Conclusions:**

Antemortem external hair contamination with synthetic cannabinoids from side-stream smoke and postmortem hair contamination with substances in putrefaction fluids can be supposed in these cases. Our findings suggest that supplementary information can indeed be gained from analyzing larvae additional to those human specimens that are typically used for toxicological analysis after extensive postmortem decomposition. Nevertheless, these results represent merely two cases, requiring in-depth studies to determine whether such findings can identify acute intoxications as possible causes of death.

## Introduction

Entomotoxicology is a subfield of forensics that applies necrophagous insects in a toxicological context [1–3]. The field has gained some interest in postmortem toxicology for detecting substances that may have caused or contributed to a drug-induced death, especially when standard toxicological procedures are limited in their application. In such cases, e.g., when processes like postmortem decomposition and animal scavenging have rendered blood and other standard postmortem specimens unavailable for toxicological analysis, the insects feeding on the corpse may be a good alternative matrix [4–6].

However, even with access to postmortem material from a highly putrefied corpse, forensic toxicologists are often confronted with analytical challenges, like matrix effects on liquid chromatographic-mass spectrometric measurements [7]. Such interferences may be less pronounced when applying necrophagous insects for toxicological analysis. In a study by Gosselin et al*.* [8], larvae produced fewer matrix effects than the putrefied human specimens during the analysis for the opioid methadone. Similarly, other entomotoxicologists reported that the analysis of insects offered more sensitive results with less interferences from decomposition by-products. Larvae may be able to metabolize those human putrefaction products that typically interfere with the analytical methods [9]. In most published studies, all drugs detected in human tissues could also be found in the insect specimens [4, 10–12]. In fact, the toxicological analysis of necrophagous insects occasionally identified substances that were not detected in the available human remains [13]. These and other findings [14, 15] suggest that the analysis of larvae has a high potential as a complementary technique to identify at least most of the substances consumed by the deceased, when human specimens are rare, highly decomposed or both.

Despite these advantages, entomotoxicological techniques do not form part of routine work in most medico-legal laboratories, still requiring the establishment of general practice guidelines and standardized analytical protocols [16]. Furthermore, most of the published data in the field resulted from experiments performed under the controlled settings of a laboratory in the form of in vitro studies, and not from its application to authentic cases [16, 17]. Of the authentic cases published, the detection of some typical drugs of abuse, such as prescription benzodiazepine drugs [6, 11], morphine [18, 19], and cocaine [10], has been reported. According to the authors’ knowledge, the detection of the so-called new psychoactive substances (NPS) in insects from real forensic cases is thus far not represented in literature.

The NPS comprise a group of drugs intended to mimic the effects of traditional drugs of abuse, such as opioids, stimulants, and cannabis. The consumption of NPS is often associated with unintentional overdoses and life-threatening poisonings. Resultantly, drug-related deaths in connection with NPS are of high forensic relevance world-wide [20–23]. Similarly, the more traditional drugs of abuse, e.g., prescription benzodiazepine drugs, still pose a high health risk, especially during polydrug use. The successful detection of drugs such as NPS and benzodiazepines in insects from decomposed human remains may thus be of great value in solving forensic cases when results from standard postmortem toxicological procedures are less reliable.

The purpose of the current investigation was to explore the potential of necrophagous larvae as a complementary matrix to identify NPS and other relevant substances in cases of extensive postmortem decomposition. A comparative analysis was performed between drugs detected in larvae and those detected in the available, putrefied human specimens from two corpses of drug-related fatalities.

## Case histories

The two cases presented here are unrelated. The deceased were discovered in their respective apartments, approximately 1 month and 1 week after cases 1 and 2, respectively, were last seen alive. Both corpses were found in June, thus during summer. The bodies were transported to the Institute of Forensic Medicine in Munich, Germany, for autopsy.

The most relevant findings of the medico-legal autopsies in both cases included the extensive postmortem decomposition, accompanied by significant defects to one of the bodies due to maggot infestation. No indications of external violence to the bodies could be identified, as far as the state of decomposition would allow such identification. Considering the case histories, death by intoxication with drugs of abuse seemed likely. Both deceased persons were known for recreational drug use, for which case 2 underwent methadone substitution therapy. Furthermore, a syringe with a needle, marihuana plants, and utensils for drug synthesis were discovered in the apartment of case 1, which further suggested a fatal intoxication with drugs of abuse.

## Materials and methods

### Postmortem sample collection

**Fig. 1:**
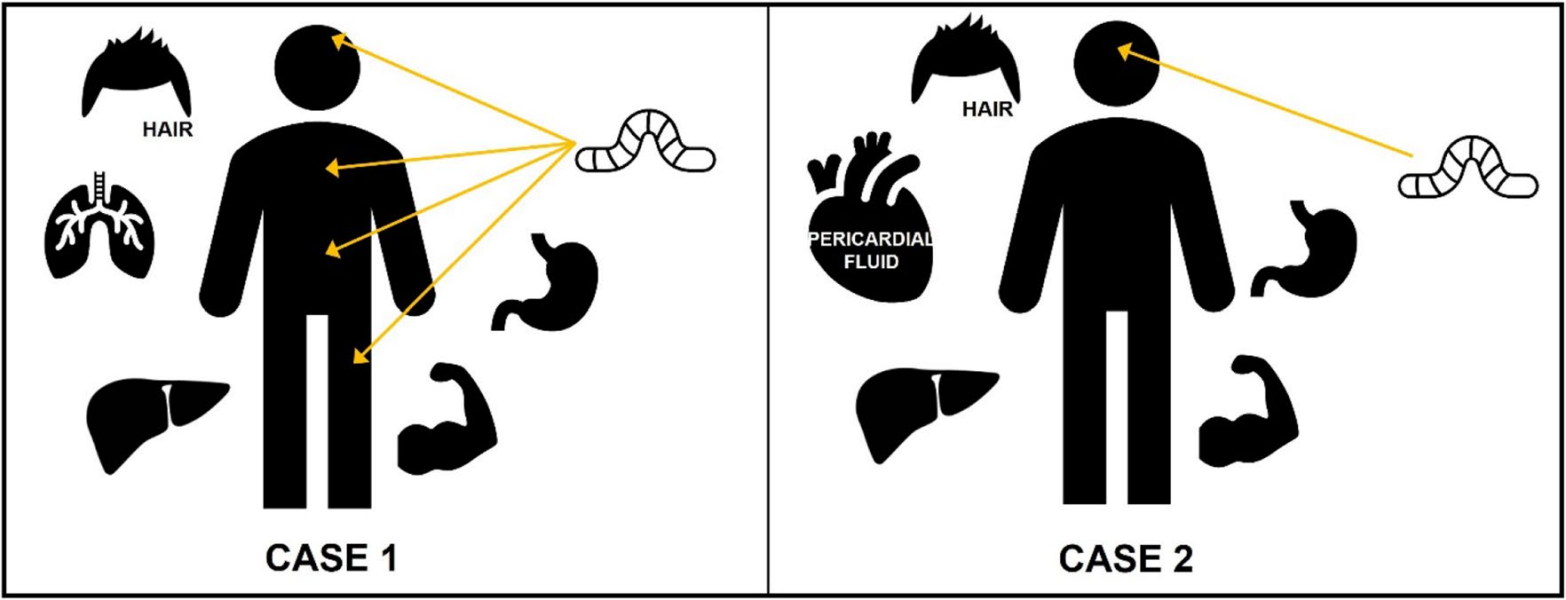
Graphical representation of the different matrices used for toxicological analysis of cases 1 and 2, respectively, including the areas from which larvae were collected for each case

A summary of all specimens taken for toxicological analysis is shown in Fig. 1. Larvae were collected in the autopsy hall from the highest colonized areas of the respective bodies. For case 1, larvae were collected separately from the stomach area, upper thigh, chest, and head, and from the left eye and the surrounding area of case 2. After collection, larvae were killed by freezing at − 20 °C for at least one day, washed with deionised water, and dried on filter paper. No blood was available from either of the two corpses because of the advanced state of decomposition. Samples for toxicological analysis were thus taken from the stomach contents, liver, skeletal muscle tissue, and hair of both decedents. Additionally, samples were taken from the lungs of case 1 and the pericardial fluid of case 2. Hair samples were taken before the first incision at autopsy, by cutting a tuft of hair with scissors as close as possible to the scalp. Larvae and human specimens were stored at − 20 °C until analysis, except hair, which was stored at room temperature.

### Chemicals and reagents

All reagents and solvents were either of analytical grade or the highest purity available. Acetonitrile (99.9%), ammonium acetate (≥ 98%), ammonium formate solution (≥ 99%), cyclohexane (≥ 99%), ethyl acetate (99.8%), and methanol (99.9%) were obtained from Sigma-Aldrich (Steinheim, Germany). Formic acid solution (98%) was purchased from Honeywell (Offenbach, Germany). Petroleum ether was purchased from CHEMSOLUTE (Renningen, Germany). Isotonic sodium chloride (NaCl) solution (0.9%, *m/v*) was obtained from Braun Melsungen AG (Melsungen, Germany). Standard substances and deuterated analogues were purchased in solution with methanol or acetonitrile from Cayman Chemical (Ann Arbor, MI, USA), Cerilliant (Austin, TX, USA), Chiron (Trondheim, Norway), LGC Standards (Wesel, Germany), and Lipomed (Arlesheim, Switzerland). MDMB-CHMICA (1 mg/mL) was purchased from Cayman Chemical. Dihydrocodeine, morphine, nordazepam, oxazepam, and tilidine (1 mg/mL each) were purchased from Cerilliant. 5F-ADB, 5F-Cumyl-PEGACLONE, 5F-PB-22, AB-CHMINACA, EAM-2201, and THJ-2201 (0.1 mg/mL each), and FUB-AMB and U-47700 (1 mg/mL each) were purchased from Chiron. AM-2201 (0.1 mg/mL), alprazolam, benzoylecgonine, bromazepam, cocaethylene, codeine, diphenhydramine, ecgonine methylester, 2-ethylidene-1,5-dimethyl-3,3-diphenylpyrrolidine (EDDP), etizolam, fentanyl, hydroxyalprazolam, melperone, methadone, norfentanyl, nortilidine, and pregabalin (1 mg/mL each) were purchased from LGC Standards. Hydroxybromazepam and lorazepam (1 mg/mL each) were purchased from Lipomed. The following deuterated analogues were purchased from Cerilliant: alprazolam-D_5_, benzoylecgonine-D_5_, bromazepam-D_4_, cocaine-D_3_, codeine-D_3_, dihydrocodeine-D_6_, ecgonine methylester-D_3_, EDDP-D_3_, fentanyl-D_5_, hydroxyalprazolam-D_5_, lorazepam-D_4_, methadone-D_3_, morphine-D_3_, nordazepam-D_5_, nortilidine·HCl-D_3_, oxazepam-D_5_, and temazepam-D_5_ (0.1 mg/mL each). The deuterated JWH-018 *N*-(5-hydroxypentyl) metabolite-D_5_ (0.1 mg/mL) was purchased from Cayman Chemical. Water was purified with an Evoquo LaboStar PRO TWF UV purification system (Günzburg, Germany).

### Sample preparation

Standard procedures were applied for the analysis of all human specimens, according to previously published methods, including the addition of a mixture of internal standards before sample extraction [24, 25]. Analogous methods were applied for preparing and analyzing the larvae.

#### Sample preparation for detecting illicit drugs, pharmaceutical substances, and NPS, including benzodiazepines, new synthetic opioids, and stimulants

Liver, skeletal muscle, lung tissue, pericardial fluid, and larvae were subjected to a protein precipitation step before analysis. Liver, muscle, and lung tissues (2.0 g each) were first homogenized with 10 mL of isotonic NaCl solution for 5 min in an ULTRA-TURRAX^®^ (IKA, Staufen, Germany). Homogenization of larvae was performed as follows: five larvae samples of case 1 (43 mg, 130 mg, 150 mg, 170 mg, and 180 mg) and three larvae samples of case 2 (45 mg, 65 mg, and 100 mg) were each placed in a separate 2 mL disposable Precellys^®^ (Bertin Technologies, Montigny-le-Bretonneux, France) reinforced vial, to which either 100 µL (samples below 100 mg) or 200 µL (all other samples) of isotonic NaCl solution were added. Five stainless steel beads (diameter: 2.8 mm) were added to each vial and the samples were homogenized in a Precellys^®^ 24 tissue  homogenizer at 4000 rpm over 30 s, analogous to the method used by Gosselin et al. [8].

For protein precipitation, 100 µL each of the liver, muscle, and lung homogenates, the total resulting volume of larval homogenate, and 100 µL of pericardial fluid were treated with 1 mL of acetonitrile, vortexed for 1 min, and centrifuged for 5 min. The supernatant was evaporated to dryness under nitrogen at 37 °C and reconstituted in 150 µL of methanolic ammonium formate solution (5 mM ammonium formate in water and methanol; 85:15, *v/v*). Stomach contents were diluted 1:10 with water, followed by a 1:1 dilution with 75 µL of methanolic ammonium formate solution (5 mM ammonium formate in water and methanol; 85:15, *v/v*).

All extracts were analyzed by liquid chromatography–tandem mass spectrometry (LC–MS/MS) and liquid chromatography–quadrupole time-of-flight mass spectrometry (LC–QTOF-MS).

#### Sample preparation for the analysis of synthetic cannabinoids

Homogenization of liver, muscle, lung tissue, and larvae was performed as described above. Six larvae samples of case 1 (16 mg, 38 mg, 140 mg, 170 mg, 180 mg, and 190 mg) and three larvae samples of case 2 (36 mg, 52 mg, and 100 mg) were prepared for the analysis of synthetic cannabinoids.

For liquid–liquid extraction, 500 µL each of liver, muscle and lung homogenates, pericardial fluid, diluted stomach contents (1:10 with water), and the total resulting volume of each of the homogenized larvae samples were treated with 1 mL of an ethyl acetate and cyclohexane mixture (1:7, *v/v*), vortexed for 1 min, and centrifuged for 5 min. The supernatant was evaporated to dryness under nitrogen at 37 °C and reconstituted in 40 µL of acetonitrile and 110 µL of methanolic ammonium formate solution (5 mM ammonium formate in water and methanol; 1:1, *v/v*). All extracts were injected into the LC–MS/MS system as described below.

#### Sample preparation of hair

An analogous method as described for previously published work was applied to prepare hair samples for analysis [26]. In brief, after washing with commercially available shampoo, the total length of each hair sample (case 1: 33 cm; case 2: 2 cm) was weighed in a plastic tube (case 1: 57 mg; case 2: 53 mg), washed with 5 mL each of petroleum ether and methanol and cut with scissors. For extraction, the hair samples were treated in an ultrasonic bath for 4 h at 50 °C, using 3 mL of methanol. The extracts were evaporated to dryness under nitrogen at 55 °C and reconstituted in 20 µL of acetonitrile and 130 µL of 2 mM ammonium acetate solution. The extracts were analyzed by LC–QTOF-MS for the detection of illicit drugs, pharmaceutical substances and NPS, including benzodiazepines, new synthetic opioids, and stimulants, and by LC–MS/MS for the detection of synthetic cannabinoids.

### Instrumentation

#### LC–MS/MS analysis for the detection of illicit drugs and pharmaceutical substances

Chromatographic separation was achieved on an Agilent 1290 Infinity instrument (Agilent Technologies, Waldbronn, Germany). One microlitre of each sample was injected onto a Zorbax Eclipse XDB-C_8_ column (4.6 × 150 mm, particle size 5 µm) (Agilent Technologies, Waldbronn, Germany). Mobile phases consisted of 5 mM ammonium formate in water with 0.1% formic acid (eluent A) and 5 mM ammonium formate in methanol with 0.01% formic acid (eluent B). The following gradient was used: 0–1 min: 10–15% B (0.5 mL/min), 1–4.7 min: 15–50% B (0.5 mL/min), 4.7–5 min: 50–51% B (0.7 mL/min), 5–10 min: 51–55% B (0.85 mL/min), 10–12 min: 55–80% B (0.95 mL/min), 12–17 min: 80–100% B (0.95 mL/min), 17–17.1 min: 100–10% B (0.95 mL/min), 17.1–18.5 min: 10% B (0.5 mL/min), and 1.5 min 10% B for re-equilibration.

Mass spectrometric analysis was performed using a QTRAP 6500 system (Sciex, Darmstadt, Germany), operated in scheduled multiple reaction monitoring (sMRM) mode, with positive electrospray ionization (ESI). Data were acquired with the Analyst 1.7.1 Software (Sciex) and processed with the MultiQuant 3.0.3 Software (Sciex).

Substances detected in liver and larvae samples were quantified by LC–MS/MS. Liver was chosen as matrix for quantification to ascertain a possible cause of death, because more reference values for toxic drug concentrations in liver are available from the literature as compared to the remaining available matrices. Quantitative data in larvae were obtained for comparison with concentrations in liver samples.

#### LC–QTOF-MS analysis for detecting illicit drugs, pharmaceutical substances, and NPS, including benzodiazepines, new synthetic opioids, and stimulants

The extracts were analyzed using an Agilent 1200 series instrument (Agilent Technologies), coupled to a TripleTOF 5600 mass spectrometer (Sciex, Concord, Ontario, Canada). The detailed method is described elsewhere [24]. Briefly, gradient elution was performed on a Zorbax Eclipse XDB-C_8_ column (4.6 × 150 mm, particle size 5 µm) (Agilent Technologies). Mobile phases consisted of 5 mM ammonium formate in water with 0.1% formic acid (eluent A) and 5 mM ammonium formate in methanol with 0.01% formic acid (eluent B). The injection volume was 10 µL and the flow rate was set at 0.85 mL/min. The total run time was 15 min.

The mass spectrometer was operated in both positive and negative ESI modes. QTOF-MS data were acquired by the Analyst TF 1.6 Software (Sciex, Darmstadt, Germany). Data processing was performed with the PeakView 2.2 Software (Sciex, Darmstadt, Germany) and MasterView 1.1 Software (Sciex).

#### LC–MS/MS analysis for the detection of synthetic cannabinoids

LC–MS/MS analysis for detecting synthetic cannabinoids was performed on an Agilent 1290 Infinity system (Agilent Technologies), coupled to a QTRAP 6500 + system (Sciex, Darmstadt, Germany), and operated in sMRM mode with positive ESI. The method is described in detail elsewhere [27]. Briefly, chromatographic separation was achieved on a Kinetex C_18_ reversed-phase column (3.0 × 50 mm, particle size 2.6 µm) (Phenomenex, Aschaffenburg, Germany). Mobile phases consisted of 2 mM ammonium acetate in 5% acetonitrile with 0.02% formic acid (eluent A) and 95% acetonitrile (eluent B). The injection volume was 10 µL and the flow rate was set at 0.4 mL/min. The total run time was 12 min.

Data were acquired with the Analyst 1.7.1 Software (Sciex, Darmstadt, Germany) and processed using the MultiQuant 3.0.3 Software (Sciex).

## Results

### Case 1

#### LC–MS/MS analysis for the detection of illicit drugs and pharmaceutical substances

The results of the LC–MS/MS analyses of case 1 are summarized in Table 1, including quantitative data from the liver and larvae samples.

**Table 1:** Substances detected by LC-MS/MS in the liver, muscle, lung tissue, stomach contents and five different larvae samples from case 1, and those substances also detected in hair by LC-QTOF-MS

	LC–MS/MS										LC–QTOF-MS
	Liver tissue [µg/kg]	Muscle tissue	Lung tissue	Stomach contents	Larvae, incl. sample weight, area of collection and substance concentrations *[µg/kg]*						Hair
					Stomach area			Thigh	Chest	Mean ± *SD*	
					Sample 1 (150 mg)	Sample 2 (180 mg)	Sample 3 (170 mg)	Sample 4 (130 mg)	Sample 5 (43 mg)		
Fentanyl	140	**✓**	**✓**	**✓**	5.9	7.4	n.d.	7.4	11	8.1 ± 2.4	**✓**
Norfentanyl	Traces	n.d.	**✓**	n.d.	n.d.	n.d.	n.d.	Traces	n.d.	–	n.d.
Tilidine	180	**✓**	**✓**	**✓**	17	11	n.d.	6.3	14	12 ± 5.8	**✓**
Nortilidine	1300	**✓**	**✓**	**✓**	54	68	n.d.	62	110	75 ± 27	**✓**
Pregabalin	79,000	**✓**	**✓**	**✓**	10,000	12,000	8,000	15,000	11,000	11,000 ± 2700	**✓**
Ecgonine methyl ester	30	**✓**	**✓**	**✓**	6.4	5.3	4.5	14	4.8	7.0 ± 4.0	n.d.
Benzoylecgonine	Traces	**✓**	**✓**	**✓**	Traces	Traces	Traces	Traces	Traces	–	**✓**
Diphenhydramine	40	**✓**	**✓**	n.d.	n.d.	n.d.	n.d.	n.d.	n.d.	–	**✓**
Cocaethylene	n.d.	n.d.	**✓**	n.d.	n.d.	n.d.	n.d.	n.d.	n.d.	–	n.d.
	LC–QTOF-MS										
U-47700	n.d.	﻿n.d.	﻿n.d.	﻿n.d.	**✓**	**✓**	**✓**	**✓**	﻿n.d.	–	**✓**
*N*-Desmethyl-U-47700	**✓**	﻿n.d.	**✓**	﻿n.d.	**✓**	**✓**	**✓**	﻿n.d.	﻿n.d.	–	**✓**
*N*,*N*-Didesmethyl-U-47700	**✓**	﻿n.d.	**✓**	﻿n.d.	﻿n.d.	﻿n.d.	﻿n.d.	﻿n.d.	﻿n.d.	–	**✓**
Etizolam	**✓**	﻿n.d.	**✓**	**✓**	﻿n.d.	﻿n.d.	﻿n.d.	﻿n.d.	﻿n.d.	–	﻿n.d.

**✓** = Detected; n.d. = not detected

Concentrations in the liver and larvae are given as the approximate amount in µg per kilogram [µg/kg]. The area of collection, the weight of each larvae sample, as well as the mean concentrations detected and standard deviations (SD) between measurements are included. Substances not included in the LC–MS/MS method, but positively identified by LC–QTOF-MS, are given below

#### LC–QTOF-MS analysis for detecting illicit drugs, pharmaceutical substances, and NPS, including benzodiazepines, new synthetic opioids, and stimulants

All substances that are not included in the LC–MS/MS method, but detected during the LC-QTOF-MS analysis, are included in Table 1.

All substances detected in the hair sample by LC–QTOF-MS, that were also detected in the other matrices by LC–MS/MS, are included in Table 1. The following substances were not detected in any of the other matrices, but were detected qualitatively in the hair sample of case 1: cocaine, norcocaine, U-51754, 6-monoacetylmorphine, dihydrocodeine, noscapine, papaverine, tramadol, carbamazepine, levomepromazine, and 3,4-methylenedioxymethamphetamine (MDMA).

#### LC–MS/MS analysis for the detection of synthetic cannabinoids

The synthetic cannabinoids detected in the different matrices originating from case 1 are summarized in Table 2.

**Table 2:** Synthetic cannabinoids detected during the LC–MS/MS analysis of hair, liver, muscle, lung, and stomach contents and six different larvae samples from case 1

	Hair	Liver, muscle, and lung tissue	Stomach contents	Larvae samples, including area of collection and weight of each sample					
				Stomach area			Thigh	Chest	Head
				Sample 1 (170 mg)	Sample 2 (190 mg)	Sample 3 (180 mg)	Sample 4 (140 mg)	Sample 5 (16 mg)	Sample 6 (38 mg)
5F-ADB	**✓**	n.d.	**✓**	﻿n.d.	﻿n.d.	﻿n.d.	﻿n.d.	﻿n.d.	﻿n.d.
5F-PB-22	**✓**	n.d.	n.d.	**✓**	﻿n.d.	**✓**	n.d.	﻿n.d.	﻿n.d.
AB-CHMINACA	**✓**	n.d.	﻿n.d.	﻿n.d.	﻿n.d.	﻿n.d.	﻿n.d.	﻿n.d.	﻿n.d.
AM-2201	n.d.	﻿n.d.	﻿n.d.	**✓**	﻿n.d.	﻿n.d.	﻿n.d.	﻿n.d.	﻿n.d.
EAM-2201	**✓**	﻿n.d.	﻿n.d.	**✓**	**✓**	**✓**	**✓**	﻿n.d.	**✓**
FUB-AMB	**✓**	﻿n.d.	**✓**	**✓**	﻿n.d.	﻿n.d.	﻿n.d.	﻿n.d.	﻿n.d.
MDMB-CHMICA	**✓**	﻿n.d.	﻿n.d.	**✓**	﻿n.d.	﻿n.d.	﻿n.d.	﻿n.d.	﻿n.d.
THJ-2201	﻿n.d.	n.d.	﻿n.d.	**✓**	﻿n.d.	﻿n.d.	﻿n.d.	﻿n.d.	﻿n.d.

**✓** = Detected; n.d. = Not detected

### Case 2

#### LC–MS/MS analysis for the detection of illicit drugs and pharmaceutical substances

A summary of all substances detected in the different matrices originating from case 2 is given in Table 3, including quantitative data from the liver and larvae samples.

**Table 3:** Substances detected by LC-MS/MS in the liver, muscle, lung tissue, and stomach contents and three different larvae samples from case 2. Those substances also detected in hair by LC-QTOF-MS are included

	*LC–MS/MS*								*LC–QTOF-MS*
	Liver tissue [µg/kg]	Muscle tissue	Pericardial fluid	Stomach contents	Larvae samples, including weight of each sample [µg/kg]				Hair
					Sample 1 (100 mg)	Sample 2 (45 mg)	Sample 3 (65 mg)	Mean ± *SD*	
Ecgonine methyl ester	Traces	**✓**	**✓**	﻿n.d.	﻿n.d.	﻿n.d.	﻿n.d.	–	﻿n.d.
Benzoylecgonine	Traces	**✓**	**✓**	﻿n.d.	﻿n.d.	﻿n.d.	﻿n.d.	–	﻿n.d.
Methadone	13,000	**✓**	**✓**	**✓**	340	250	320	310 ± 49	**✓**
*EDDP	2000	**✓**	**✓**	**✓**	890	530	700	710 ± 170	**✓**
Morphine	130	**✓**	**✓**	**✓**	58	38	30	42 ± 14	**✓**
Codeine	n.d.	﻿n.d.	**✓**	**✓**	﻿n.d.	﻿n.d.	﻿n.d.	–	**✓**
Dihydrocodeine	﻿n.d.	﻿n.d.	﻿n.d.	**✓**	﻿n.d.	﻿n.d.	﻿n.d.	–	﻿n.d.
Nordazepam	150	**✓**	**✓**	**✓**	86	44	37	55 ± 26	**✓**
Oxazepam	7000	**✓**	**✓**	**✓**	9000	5100	5900	6800 ± 2200	**✓**
Lorazepam	1100	**✓**	**✓**	**✓**	1400	540	670	870 ± 470	**✓**
Bromazepam	1000	**✓**	**✓**	**✓**	670	200	300	390 ± 240	**✓**
Hydroxybromazepam	Qualitative	**✓**	**✓**	﻿n.d.	Traces	Traces	Traces	–	﻿n.d.
Alprazolam	550	**✓**	**✓**	**✓**	40	20	29	30 ± 10	**✓**
Hydroxyalprazolam	30	﻿n.d.	**✓**	﻿n.d.	Traces	Traces	Traces	–	﻿n.d.
Pregabalin	57,000	**✓**	**✓**	**✓**	34,000	28,000	22,000	28,000 ± 5800	**✓**
Paracetamol	240	**✓**	**✓**	**✓**	﻿n.d.	﻿n.d.	﻿n.d.	–	﻿n.d.
Melperone	10	**✓**	**✓**	﻿n.d.	Traces	Traces	Traces	–	﻿n.d.
	LC-QTOF-MS								
Noscapine	**✓**	**✓**	**✓**	**✓**	**✓**	**✓**	**✓**	–	**✓**
Papaverine	﻿n.d.	﻿n.d.	﻿n.d.	**✓**	﻿n.d.	﻿n.d.	﻿n.d.	–	**✓**

✓ = Detected; n.d. = Not detected

Concentrations in liver and larvae are given as the approximate amount in µg per kilogram [µg/kg]. The weight of each larvae sample, as well as the mean concentrations detected, and standard deviations (SD) are included. Substances not included in the LC-–MS/MS method, but positively identified by LC–QTOF-MS, are given below

^*^EDDP = 2-ethylidene-1,5-dimethyl-3,3-diphenylpyrrolidine

#### LC–QTOF-MS analysis for detecting illicit drugs, pharmaceutical substances, and NPS, including benzodiazepines, new synthetic opioids, and stimulants

All substances that are not included in the LC–MS/MS method, but were detected during the LC–QTOF-MS analysis, are included in Table 3.

All substances detected in the hair sample by LC–QTOF-MS that were also detected in the other matrices during LC–MS/MS are included in Table 3. The following substances were detected qualitatively in the hair sample of case 2, but were not detected in any of the other matrices: 7-aminoclonazepam, zopiclone, and pipamperone.

#### LC–MS/MS analysis for detecting synthetic cannabinoids

The synthetic cannabinoids detected in the matrices originating from case 2 are presented in Table 4.

**Table 4:** Synthetic cannabinoids detected during the LC–MS/MS analysis of hair, liver, muscle, pericardial fluid, and stomach contents and three different larvae samples from case 2

	Hair	Liver, muscle, and pericardial fluid	*Stomach contents*	Larvae samples, including weight of each sample		
				Sample 1 (100 mg)	Sample 2 (36 mg)	Sample 3 (52 mg)
5F-ADB	**✓**	n.d.	n.d.	n.d.	n.d.	n.d.
5F-Cumyl-PEGACLONE	**✓**	n.d.	**✓**	**✓**	**✓**	**✓**
5F-PB-22	**✓**	n.d.	n.d.	n.d.	n.d.	n.d.
MDMB-CHMICA	**✓**	n.d.	n.d.	n.d.	n.d.	n.d.

**✓** = Detected; n.d. = Not detected

## Discussion

The extensive postmortem decomposition of both cases complicated the ascertainment of a reliable cause of death by autopsy. The case histories pointed toward drug-associated fatalities.

During toxicological analysis, the quantification of postmortem samples is always subject to various postmortem artifacts, especially after significant postmortem degradation had occurred. The concentrations in the tissues given are thus merely approximations of the true values [28, 29].

**Case 1:** In the literature, liver fentanyl concentrations of fatal intoxication cases range from 5.9 to 203 µg/kg [30]. Case 1’s liver fentanyl concentration of approximately 140 µg/kg lies well within this range, thus making an intoxication with fentanyl likely to be the leading cause of death. The detection of the fentanyl metabolite  norfentanyl in trace amounts in the liver sample suggests a time of death shortly after the deceased last consumed fentanyl. Furthermore, the additional central nervous system depressing effects by the opioid tilidine, the new synthetic opioid U-47700, the antiepileptic drug pregabalin, the benzodiazepine etizolam, and possibly also the synthetic cannabinoids may have contributed to this fatality.

It was interesting to note that the liver, muscle, and lung tissues of case 1 tested negative for synthetic cannabinoids, whereas the synthetic cannabinoids 5F-ADB and FUB-AMB were detected in the stomach contents. On the other hand, altogether six synthetic cannabinoids could be detected in the larvae. These findings are potentially significant, because liver and perhaps muscle are usually applied as an alternative matrix during routine postmortem casework where blood is absent. A similar distribution pattern for 5F-ADB in human tissues was found in a case study by Hasegawa et al*.*, for which the authors suggested that the significantly lower concentrations in solid tissues may have resulted from the short period between inhaling the synthetic cannabinoid by smoking and the time of death. Resultantly, only small amounts of 5F-ADB could be incorporated into bodily tissues via the lungs during this short timespan [31]. Furthermore, the rapid metabolism of synthetic cannabinoids after consumption may also play a role [32, 33]. Our finding, and perhaps also the positive findings in the stomach contents of the case published by Hasegawa et al*.* [31], may have resulted at least partially from swallowing saliva that became contaminated with the synthetic cannabinoids during smoking shortly before death. This might explain our inability to detect 5F-ADB in any of the larvae samples, and the detection of FUB-AMB in only one larvae sample, despite positive findings in the stomach contents. Likewise, other authors reported comparatively high concentrations of synthetic cannabinoids in stomach contents [34, 35]. Others demonstrated the preferable distribution of these drugs into adipose tissues, in combination with low concentrations in soft tissues, like liver.

In their study on the distribution patterns of two synthetic cannabinoids after intravenous administration to pigs, Schaefer et al*.* [36] found that the highest perimortem concentrations were observed at 6 h after drug administration in abdominal and perirenal adipose tissue for JWH-210, and lungs, abdominal adipose tissue, perirenal adipose tissue, and bile fluid for RCS-4. The lowest perimortem concentrations of the two parent compounds were observed in the dura, lung and liver, and heart, liver, spleen, and dura, respectively. These results and also findings by others [32] suggest that adipose tissue might be one of the best alternative postmortem matrices, or even the matrix of choice for detecting synthetic cannabinoids, even when death occurred soon after consumption. However, it is unclear whether similar distribution patterns can be expected after pulmonary administration and possible postmortem redistribution of the drugs had occurred. Schaefer et al*.* did publish follow-up studies on time- and temperature-dependent postmortem concentrations after pulmonary administration of JWH-210 and RCS-4. However, these studies did not include adipose tissue as a sample matrix [37, 38]. Regardless, adipose tissues are not typically analyzed during routine postmortem toxicology analyses. Because of its complexity, sample preparation for extracting and detecting drugs from such tissues can be challenging. On the other hand, the larvae feeding on the corpse also consumes fat, after which the substances therein might be absorbed into and bioaccumulate in larval tissues. Furthermore, it has been suggested that human postmortem degradation products that typically interfere with chromatographic analyses might be digested and excreted by larvae [9], suggesting that larvae might be a better alternative to adipose tissue for toxicological analysis. More detailed studies are required to confirm these hypotheses.

Except for 5F-ADB and AB-CHMINACA, all synthetic cannabinoids detected in the hair were also identified in the larvae. In fact, two additional synthetic cannabinoids (AM-2201 and THJ-2201) that were not present in any of the other matrices, including hair, could be identified in one larvae sample. This may have resulted from an accumulation of these substances in adipose tissue, likely combined with the area from which the larvae from this one sample were feeding, and the larvae’s developmental stage at the time of collection. These arguments may also apply to the detection of six different synthetic cannabinoids only in this one sample (see Table 2). Thus, our findings by no means confirm an acute intoxication with synthetic cannabinoids close to death, and further investigations are necessary to evaluate the time-dependent distribution and accumulation of these substances in human specimens and larvae.

The new synthetic opioid U-47700 was also detected exclusively in larvae and hair, although only in trace amounts in the larvae. Despite its absence in all other matrices, a prior consumption could be confirmed by detection of the two metabolites *N*-desmethyl-U-47700 and *N,N*-didesmethyl-U-47700 in the liver, lungs, and hair. The main metabolite *N*-desmethyl-U-47700 was also detected in the larvae, but not the more polar metabolite *N,N*-didesmethyl-U-47700. These findings are in accordance with results of another research group on the distribution patterns of U-47700 and its main metabolite. Nordmeier et al*.* reported that, following intravenous application of U-47700 to pigs, lung, and liver tissues contained comparatively higher concentrations of *N*-desmethyl-U-47700, whereas higher ratios of the parent compound were found in adipose tissues [39]. As discussed for the synthetic cannabinoids, adipose tissues are not routinely analyzed during toxicological analyses, but are likely consumed by the larvae. Considering its high potency, U-47700 might have contributed significantly to this fatality [25].

The detection of the semi-synthetic opioid analgesic dihydrocodeine, the opiate alkaloids papaverine and noscapine, the synthetic opioid tramadol, the new synthetic opioid U-51754, the anticonvulsant drug carbamazepine, the neuroleptic drugs quetiapine and levomepromazine, the synthetic cannabinoid AB-CHMINACA, and the local anesthetic drug lidocaine exclusively in the hair sample suggests the last consumption of these substances a significant time before death. A relevant influence on the death of case 1 by these substances is thus not expected. The detection of 6-monoacetylmorphine, cocaine, and its metabolite norcocaine only in hair may be explained at least partially by their short elimination half-lives and/or postmortem instability in other matrices [28, 40]. The detection of 6-monoacetylmorphine in combination with papaverine and noscapine suggests a preceding consumption of heroin.

The detection of tilidine and its metabolite nortilidine, as well as pregabalin and the cocaine metabolites ecgonine methyl ester and benzoylecgonine in larvae was well comparable to their detectability in other matrices. In contrast, the benzodiazepine etizolam and the antihistamine diphenhydramine were not detected in the larvae. Differences in tissue distribution and accumulation patterns in humans and larvae, compared to the more lipophilic substances like the synthetic cannabinoids or U-47700, might explain why these drugs were not detectable in the larvae, nor in several other investigated human specimens.

**Case 2:** The leading cause of death of case 2 was likely an intoxication with the opioid methadone. According to the case history, the deceased underwent methadone substitution therapy for drug abuse. The concentration of approximately 13 mg of methadone per kilogram liver (13,000 µg/kg) lies above the range (1800–7500 µg/kg) given in the literature for fatal methadone-related intoxication cases [30]. A synergistic effect with the other central nervous system depressing drugs (e.g., the benzodiazepines) is expected.

The synthetic cannabinoid 5F-Cumyl-PEGACLONE was detected in all larvae samples, as well as stomach contents. Contamination of stomach contents through swallowing synthetic cannabinoid containing saliva during smoking shortly before death may also be possible for case 2. However, as with case 1, no synthetic cannabinoids could be detected in liver and muscle tissues of case 2, nor in the pericardial fluid. Also here, a short timespan between smoking and death, and the rapid metabolism of these drugs may be possible explanations [31–33].

Nearly all substances detected in one or more of the human specimens were also detected in the larvae. The cocaine metabolites ecgonine methyl ester and benzoylecgonine were detected in all human specimens, except for stomach contents, but not in any of the larvae samples. However, both substances were detected in case 1. The undetectability in case 2 might be explained by the distribution pattern of ecgonine methyl ester and benzoylecgonine in the human body and thus also the area from which the larvae were feeding. Larvae were only present in the area surrounding case 2’s left eye, which likely did not contain these substances, whereas larvae could be collected from several areas from case 1. Likewise, the rather more hydrophilic substances codeine, dihydrocodeine, the non-opioid analgesic paracetamol, and papaverine were also not detected in the larvae from case 2.

The detection of the non-benzodiazepine hypnotic drug zopiclone and the benzodiazepine metabolite and degradation product 7-aminoclonazepam exclusively in hair may be explained by their poor physicochemical stability in the other matrices. The last consumption of zopiclone and clonazepam a significant time before death is also possible. The latter may also apply to the detection of the synthetic cannabinoids 5F-ADB, 5F-PB-22, and MDMB-CHMICA only in hair.

During the toxicological investigation of various matrices originating from the two cases presented here, some substances were detected in larvae, but not in those matrices routinely analyzed after extensive postmortem decomposition (e.g., liver). Some of the most unexpected results include the identification of synthetic cannabinoids and U-47700 in larvae, whereas these substances were not detected in most of the human tissues that were available for toxicological analysis. Furthermore, some substances, like etizolam, were not detectable in larvae.

Several factors may influence the detectability of substances in larvae. The area of feeding and the developmental stage of the larvae [6, 10, 41] determine which substances they consume and the extent of their bioaccumulation in larvae. Moreover, the drug’s pharmacokinetic behavior in both humans and larvae, as well as its physicochemical properties should be considered. At present, not much is known about the metabolism and accumulation of drugs in necrophagous larvae [42]. As with humans, if a drug’s rate of absorption exceeds its rate of elimination, it should bioaccumulate in the larval tissues. In a study by Wilson et al*.* [43], the authors presumed that the rapid elimination of paracetamol by larvae might explain their inability to detect the substance in the larvae [6]. Hydrophilic substances are believed to be excreted rapidly through the so-called Malphigian tubules, whereas other molecules follow a more complex route. Bourel et al*.* [44], for example, discovered by immunochemical investigation that morphine accumulated in the area between the endocuticle and exocuticle of the larva, whereas the digestive tract tested negative for morphine. This suggests that, depending on their physicochemical properties, different drugs may be incorporated into certain areas of the larvae, while others are eliminated much faster. The excellent detectability of the synthetic cannabinoids and U-47700 in our cases suggests that the substances’ lipophilic nature contributes to higher bioaccumulation in human adipose tissue and/or larval tissues. Such bioaccumulation in combination with the reported rapid metabolism of synthetic cannabinoids would also explain their undetectability in most human tissues [32, 33].

Another aspect to be considered when comparing the detectability of substances between human specimens and larvae is potential differences in matrix effects. The interferences from human tissues on the analytical techniques may differ significantly from those originating from larvae. Earlier studies have shown that the analysis of larvae can produce more sensitive results than decomposed human specimens [8, 11]. The ability of larvae to metabolize and excrete interfering putrefaction products originating from the corpse may play a role in this regard [9].

In both cases presented here, the quantification in larvae produced high variability in substance concentrations between different samples. Moreover, no correlation could be found between concentrations in liver samples and the larvae. This was expected, since drug quantities in larvae are also highly dependent on several factors, including the larvae’s developmental stage at the time of sampling [45] and the area of the corpse it was feeding on [3, 6, 10, 12, 46]. Thus, the application of larvae is of limited quantitative value to estimate whether lethal dosages of a substance were consumed by the deceased. Nevertheless, the toxicological analysis of highly decomposed human specimens are faced with similar challenges and the detection of some substances in such materials may even be entirely hindered [29, 47, 48]. Considering the high potency of many recreational drugs, even their qualitative detection might help elucidate the cause of death when other options are limited. Therefore, larvae may be a good additional matrix for toxicological analysis of extensively decomposed corpses.

In light of our findings, we recommend preserving larvae in all postmortem cases where they may be present on and inside the corpse. This would allow the possibility to use them as an additional matrix for toxicological investigation when the conventional methods produce unsatisfying results. It is, however, important to consider that enough material is collected. From our experience, approximately 100–200 mg of larval material for a single extraction provided the most promising results. Nevertheless, practical aspects for the application of larvae in forensic casework remain a challenge that require the standardization of sample preparation and analysis guidelines [2, 16]. Based on the results obtained during this study, pre-validation experiments and the validation of methods to detect specific drugs in larvae are currently underway.

## Conclusions

The absence of a substance in conventionally investigated human specimens does not necessarily imply its absence in the corpse. Some substances, e.g., synthetic cannabinoids, undergo rapid metabolism and have a high affinity for adipose tissue [32, 33, 36]. Adipose tissues are not typically analyzed during standard toxicological analyses, but may be ingested by and the substances therein bioaccumulated in the insects feeding on the corpse. More detailed studies on time-dependent distribution patterns of specific drugs are required to conclude whether their detection in larvae can be used to identify an acute intoxication as cause of death.

In conclusion, our preliminary results suggest that the application of larvae as a complementary matrix in cases of extensive postmortem decomposition may indeed produce additional toxicological information. Contrary to hair analyses, entomotoxicological methods may not only be useful to identify drugs consumed an extended period before death, but possibly also sooner. Furthermore, external contamination with synthetic cannabinoids via side-stream smoke and with substances in putrefaction fluids should also be considered when applying hair as matrix for toxicological analysis. These challenges are not relevant when applying larvae.

Considering the current effect of NPS and other potent substances on drug-related fatalities [22, 23, 49], an additional option for their detection may be of great value in solving forensic casework, when the reliability of standard toxicological procedures is questionable. Although entomotoxicological analyses do not allow an estimation of the dose of drug exposure, they may be valuable as a complementary method for qualitative identification [10]. Larvae can even act as an alternative matrix when no other biological specimens are available, e.g., due to skeletonisation or animal scavenging of human remains. Even the qualitative detection of potent substances, like some synthetic cannabinoids and new synthetic opioids, may help the forensic toxicologist to ascertain the cause of death when all other possibilities are limited.

It should nevertheless be kept in mind that the results presented here originate from merely two cases. This work can thus not be considered a representative sample to illustrate the potential of entomotoxicological methods in routine laboratory work. Furthermore, the lack of standardized methods for the extraction and toxicological analysis of substances from larvae further underlines the necessity of future research on this subject [16]. Validation experiments will follow.
